# A Herbivore Knows Its Patch: Luderick, *Girella tricuspidata*, Exhibit Strong Site Fidelity on Shallow Subtidal Reefs in a Temperate Marine Park

**DOI:** 10.1371/journal.pone.0065838

**Published:** 2013-05-31

**Authors:** Adrian M. Ferguson, Euan S. Harvey, Matthew D. Taylor, Nathan A. Knott

**Affiliations:** 1 The UWA Oceans Institute and School of Plant Biology, Faculty of Natural and Agricultural Sciences, The University of Western Australia, Crawley, Western Australia, Australia; 2 Institute for Conservation Biology and Environmental Management, School of Biological Sciences, University of Wollongong, Wollongong, New South Wales, Australia; 3 NSW Department of Primary Industries, Port Stephens Fisheries Institute, Taylors Beach, New South Wales, Australia; 4 NSW Department of Primary Industries, Jervis Bay Marine Park, Huskisson, New South Wales, Australia; University of Western Australia, Australia

## Abstract

Understanding movement patterns, habitat use and behaviour of fish is critical to determining how targeted species may respond to protection provided by “no-take” sanctuary zones within marine parks. We assessed the fine and broad scale movement patterns of an exploited herbivore, luderick (*Girella tricuspidata*), using acoustic telemetry to evaluate how this species may respond to protection within Jervis Bay (New South Wales, Australia). We surgically implanted fourteen fish with acoustic transmitters and actively and passively tracked individuals to determine fine and broad scale movement patterns respectively. Eight fish were actively tracked for 24 h d¯^1^ for 6 d (May 2011), and then intermittently over the following 30 d. Six fish were passively tracked from December 2011 to March 2012, using a fixed array of receivers deployed across rocky reefs around the perimeter of the bay. Luderick exhibited strong site fidelity on shallow subtidal reefs, tending to remain on or return consistently to the reef where they were caught and released. All eight fish actively tracked used core areas solely on their release reef, with the exception of one fish that used multiple core areas, and four of the six fish passively tracked spent between 75 to 96% of days on release reefs over the entire tracking period. Luderick did move frequently to adjacent reefs, and occasionally to more distant reefs, however consistently returned to their release reef. Luderick also exhibited predictable patterns in movement between spatially distinct daytime and night-time core use areas. Night-time core use areas were generally located in sheltered areas behind the edge of reefs. Overall, our data indicate luderick exhibit strong site fidelity on shallow subtidal reefs in Jervis Bay and suggests that this important herbivore may be likely to show a positive response to protection within the marine park.

## Introduction

The large-scale movement and migratory behaviour of many species of fish has often been used as an argument to suggest that “no-take” sanctuary zones within marine parks may be inappropriate for the protection of targeted species from fishing [1]. Many species do migrate over large distances (in some cases over thousands of kilometres annually) to feed or reproduce [2], [3], however the conventional view that fish are too mobile to benefit from protection is being challenged [4], [5]. In many instances, this is due to technological advances in our ability to track fish leading to a greater understanding of movement patterns.

Research on coral reef fish has shown that relatively sedentary species can respond rapidly to protection [6], [7] and there is increasing evidence demonstrating the value of sanctuary zones in protecting highly mobile pelagic species [7], [8], [9]. Similarly, in temperate latitudes, less mobile fish [10] and other species previously presumed to be too mobile to benefit from protection have responded strongly to spatial closures within marine parks [11], [12], [13]. For example, snapper, *Pagrus auratus*, have been shown to take up long term residency on reefs, with higher densities within protected areas, despite initial views that the species was too mobile to benefit from protection [11], [12], [13].

Sanctuary zones have been demonstrated to be effective at protecting targeted species from fishing [6], [7], [14], although the factors that lead to adequate protection may be complex. Protection of key habitat and feeding and breeding grounds, variation in life history and fish behaviour may all determine how well a species responds to protection, although in general, sanctuary zones can only be effective if they protect a significant portion of the home range and life cycle of species that reside within them [15].

Often sanctuary zones are perceived solely as a fisheries management tool and thus many of the studies of fish movements within marine parks have focussed on species valuable to commercial and recreational fisheries [9], [11], [12], [13], [16], [17], [18]. However, some of the main objectives of sanctuary zones are to conserve biodiversity and maintain ecological processes, rather than to operate primarily as a fisheries management tool. Therefore, it is surprising that little research has been carried out on the movement patterns of herbivorous species within marine parks, given their likely significance in maintaining important ecological processes, such as algal grazing [19], [20].

Luderick, *Girella tricuspidata*, is a primarily herbivorous species [21], [22], commonly found on near shore rocky reefs and within estuaries along the eastern and southern coastline of Australia and around the North Island of New Zealand [19], [23], [24]. They are considered to play an important ecological role due to their large biomass and associated grazing on rocky reefs [19], [25]. Luderick are also exploited by both recreational [24], [26], [27] and commercial [27], [28] fisheries in south-eastern Australia. The combined recreational and commercial catch in New South Wales (NSW) alone is between ∼700 and 1000 t annually [27], [29], [30] and the current exploitation status of luderick in NSW is fully fished [29]. Commercial beach hauling for luderick also takes place within Jervis Bay, NSW, however specific statistics on annual catch rates are unavailable. This significant level of exploitation, combined with the potentially important role of luderick in ecosystem functioning, makes it an ideal model species for which to quantify movement patterns and assess how it may respond to protection provided by sanctuary zones.

Luderick are considered to be highly mobile [31] and exhibit poor site fidelity [24]. Primarily, this is due to the relatively large movements they can undertake and their temporal variability on rocky reefs [24], [31]. Limited empirical data, however, exists on their movement patterns and indicate that movements are complex and involve uncertainties. Mark-recapture experiments conducted in NSW showed that tagged luderick moved distances up to 450 km from their point of release, travelling in a predominantly northerly direction along the coast [27], [32], [33]. Gray et al. [27], [31] suggested that some movements may be related to pre-spawning migrations. Luderick are generally assumed to spawn in the coastal zone, along surf beaches and near the entrance to estuaries during the austral winter, and later at higher latitudes [27], [31]. Despite these relatively large movements luderick may also remain within the same area for long periods of time. The majority of tagged luderick recaptured during the mark-recapture experiments [27], [32], [33] were caught within the same estuary in which they were released, in some instances nearly two years later, indicating that luderick were probably residing within those estuaries. Little, however, is known of their movements within these estuaries. A limitation of mark recapture experiments is that they provide little information on the fine scale movement patterns of individuals as movements can only be inferred between the point of release and recapture, and results can be biased by the temporal and spatial distribution in sampling effort. Hence, luderick may exhibit a complex range of behaviours associated with partial migration, i.e. “the phenomenon of coexisting groups exhibiting migratory and resident behaviour within the same population” [34], which is more common among marine fishes than previously recognised [16], [17], [34], [35]. Understanding the potentially complex movement patterns of luderick, and fish in general, and how they relate to the design of marine parks will provide necessary information in order to assess their potential response to protection.

The major aim of this study was to describe and quantify short-term, fine-scale movement patterns in combination with longer-term, broad-scale movement patterns of luderick using acoustic telemetry. Primarily, we were interested in the level of site fidelity, if any, luderick exhibit on shallow subtidal reefs and the frequency of movements between reefs. Secondly, our objective was to describe general movement patterns of luderick, including habitat associations and diel movements. We carried out this study within Jervis Bay, NSW, which is encompassed by both a state and Commonwealth marine park, in order to provide an initial assessment of the likelihood of sanctuary zones within the marine park providing significant protection for this species.

## Materials and Methods

### Study area

**Figure 1:**
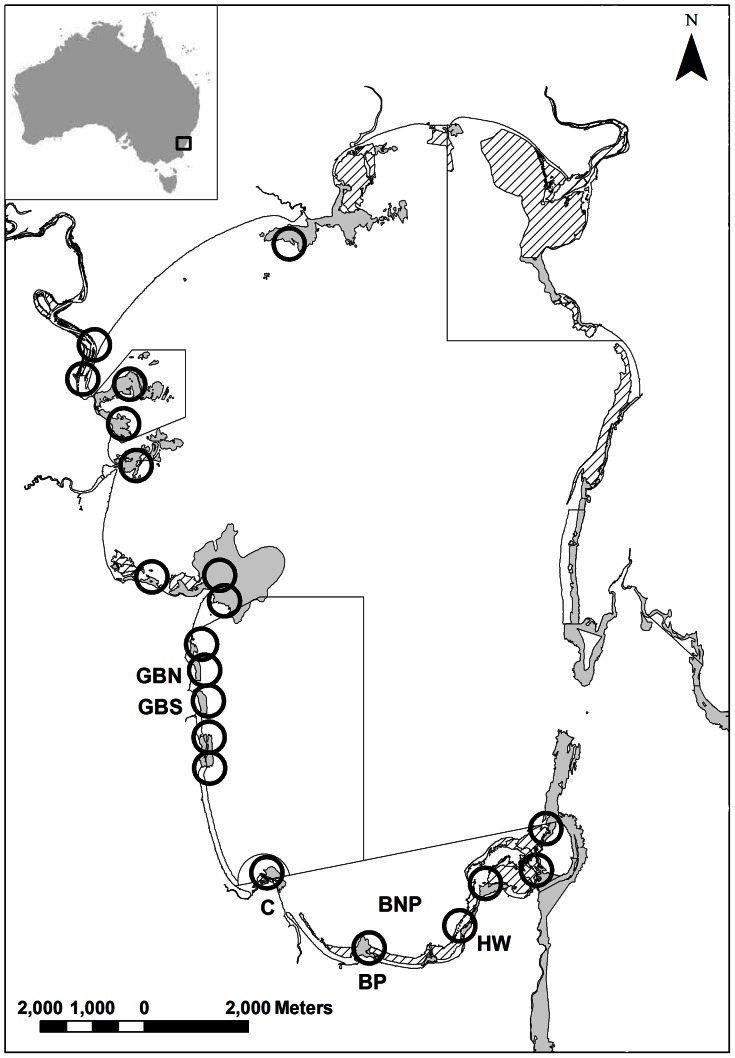
Map of Jervis Bay, New South Wales, where the study was conducted. Luderick, *Girella tricuspidata*, were caught and released at 2 sites within a sanctuary zone (GBN  =  Greenfields Beach North and GBS  =  Greenfields Beach South) and 2 sites within Booderee National Park (BNP) (BP  =  Bristol Point and HW  =  Hole in the Wall). Boxed areas, except for BNP, represent sanctuary zones (no fishing allowed). The semi-circle area at C, (Creswell), represents a special purpose zone and the site of an artificial harbour. Empty circles represent receiver locations and a 300 m detection area. Rocky reef (light grey) and seagrass (hatch) areas are shown. Inset map of Australia is also shown.

The study was conducted in Jervis Bay (35°8’S 150°43’E) during May to June 2011 and December 2011 to March 2012. Jervis Bay is a large, relatively pristine embayment in southern NSW, covering approximately 160 km^2^, and forms the central area of the Jervis Bay Marine Park. The Jervis Bay Marine Park is a multiple-use park zoned for various activities, including recreational and commercial fishing, with 20% of the park designated as “no-take” sanctuary zones, 72% as habitat protection zones and 8% as general use zones. Jervis Bay also contains Commonwealth waters of Booderee National Park (BNP) which allows recreational line fishing only. Jervis Bay has a mosaic of rocky intertidal and subtidal reefs and seagrass beds interspersed within intertidal and subtidal soft-sediments (Fig. 1). Oceanic conditions within the bay largely reflect those of adjacent coastal waters, although water quality can be affected by freshwater flows which drain into the embayment from small estuaries, particularly during large rainfall events.

### Acoustic tagging procedure

Luderick were caught on shallow subtidal reefs using hook and line and placed in a holding tank containing fresh seawater which was aerated. To compare movement patterns of luderick in areas protected from fishing and areas where fishing was allowed, fish were caught at 2 sites within a sanctuary zone and 2 sites within BNP (Fig. 1).

Two types of acoustic transmitter were used and these were surgically implanted in 14 fish, ranging between 265 to 349 mm total length (Table 1). Vemco (Amirix Systems, Halifax, Nova Scotia, Canada) V9-1L continuous transmitters (9 mm diameter, 24 mm length, 3.6 g weight in air, ∼ 37 d battery life) were implanted in 8 fish which were actively tracked to determine fine-scale movements. Continuous transmitters transmitted a series of pings, detected between 63–84 kHz, at a fixed rate suitable for real time tracking. A unique combination of ping series and frequency allowed identification of individual implanted fish. Vemco V9-2L coded transmitters (9 mm diameter, 29 mm length, 4.7 g weight in air, 738 day battery life) were implanted in 6 fish which were passively tracked to determine broad scale movements. Coded transmitters transmitted pings at 69 kHz that were infrequent and random about a nominal delay of 180 seconds. The ping series for each transmitter included an ID number which allowed identification of individual implanted fish. Transmitters weighed no more than 1.6% of the total body mass of individual fish.

**Table 1:** Summary data for luderick, *Girella tricuspidata*, tracked using acoustic telemetry in Jervis Bay, New South Wales.

Fish ID	Total length (mm)	Catch-release date	Catch-release reef	Tracking type	# days tracked
1	278	26/05/2011	BP (BNP)	Active	10 (13)
2	288	30/05/2011	HW (BNP)	Active	11 (11)
3	283	26/05/2011	BP (BNP)	Active	11 (13)
4	310	30/05/2011	HW (BNP)	Active	11 (11)
5	272	02/06/2011	HW (BNP)	Active	4 (8)
6	327	02/06/2011	HW (BNP)	Active	7 (8)
7	276	02/06/2011	HW (BNP)	Active	7 (8)
8	265	02/06/2011	HW (BNP)	Active	5 (8)
9	342	22/12/2011	GBN (sz)	Passive	73 (97)
10	340	22/12/2011	GBN (sz)	Passive	53 (97)
11	314	22/12/2011	GBS (sz)	Passive	79 (97)
12	349	22/12/2011	GBS (sz)	Passive	92 (97)
13	333	22/12/2011	BP (BNP)	Passive	41 (99)
14	305	22/12/2011	BP (BNP)	Passive	95 (99)

BP  =  Bristol Point, HW  =  Hole in the Wall, GBN  =  Greenfields Beach North, GBS  =  Greenfields Beach South. BNP  =  Booderee National Park, sz  =  sanctuary zone (no fishing allowed). For number of days tracked, ()  =  number of possible detection days.

Before fish were surgically implanted with acoustic transmitters they were allowed to rest in the holding tank for ∼15 min. to recover from the stress of capture. Fish were then anaesthetised in a separate holding tank containing 15 mg L¯^1^ AQUI-S®. Once anaesthetised, fish were placed side down on a measuring board and a 10–15 mm incision was made in the ventral surface of the fish, adjacent to the pectoral fin and towards the rear of the peritoneal cavity. Scales dislodged during the incision were removed from the wound. Transmitters were immersed in povidone-iodine antiseptic (Betadine®) to prevent infection, before being inserted into the cavity. The incision was sutured (Ethicon®; coated Vicryl braided sutures; needle reference SH; suture size 3–0) using 1 to 2 surgical knots, and a broad spectrum antibiotic (Engemycin®) was injected into the peritoneal cavity at a dose of 3 mg kg¯^1^ body weight.

After surgery, fish were returned to the original holding tank and cradled backwards and forwards through aerated seawater to increase irrigation past the gills and aid recovery. Fish were monitored until recovery, which involved actively swimming in an upright position (which generally occurred within <15 min.). All fish recovered from surgery and following recovery fish were released at their point of capture.

### Active tracking

To quantify fine-scale movement patterns of luderick fish were actively tracked, shortly after release, from a motorised boat using a receiver and directional hydrophone (Vemco VR100 and VH110 respectively) during May to June 2011. Preliminary trials indicated that the effective reliable range in which continuous transmitters could be detected unobstructed across open ground was ∼300 m. Fish were actively tracked for 24 h d¯^1^ for the first six days post release, and then predominantly tracked between dawn and dusk (initial observations indicated fish were most active during this period) for a further five days, chosen at random, for the remaining battery life of the transmitters (∼37 d). Fish were tracked sequentially and once a series of pings was detected, indicating a fish was in range, the signal was followed until the pings were strongest and a waypoint was recorded using a GPS (Garmin® GPS 60) to mark the fish’s position. A waypoint was not recorded unless the decibel (db) reading displayed on the receiver was consistently ≥70 db. Preliminary trials indicated positional accuracy was typically ∼3 m. Over the course of the tracking, fish were also chosen at random and tracked continuously over a 1 to 2 h period, with a waypoint recorded every 10 min., to determine finer-scale movements.

### Passive tracking

To determine broad scale movement patterns of luderick, fish were passively tracked during December 2011 to March 2012. An array consisting of 20 receivers (Vemco VR2W) deployed on the majority of rocky reefs around the perimeter of Jervis Bay and also at the entrance to the largest estuary flowing into the bay, was used to determine the frequency of movements between shallow subtidal reefs and across marine park management zones (Fig. 1). Receivers were separated by a minimum distance of 500 m, and placed no further than 300 m from the shoreline, as preliminary trials indicated that the maximum reliable range that transmitters could be detected unobstructed across open ground was ∼300 m. Receivers were deployed in between 3 to 8 m water depth and kept in place using a mooring system, consisting of a length of 16 mm nylon rope attached to a large sub-surface buoy and weighed down using one or two, 1 m lengths of steel railway track (53 kg each) placed on the sea floor. The receiver was cable tied upright to the length of rope approximately 2 m above the sea floor. Receivers were retrieved after 3 months and the data downloaded and analysed. To determine whether there was a difference in the effectiveness of receivers to detect transmitter signals between the day (0601 to 1800 h) and night (1801 to 0600 h) a reference transmitter was deployed ∼100 m from receivers at two sites, Greenfields Beach North and Bristol Point (Fig. 1).

### Data analysis

For the active tracking data (fine-scale movements) we quantified space utilisation distribution for each fish using the kernel density function within the spatial analyst extension in ArcGIS v.10. Time was used as the density variable to estimate space utilisation distribution. We created contour lines representing the 90% and 50% by volume contours. Home range and core use areas were defined as the areas within the 90% and 50% contours respectively. Linear regression was used to test for a relationship between fish size and both home range size and total core use area, and between home range size and total core use area. The 50:90% ratio was calculated for each fish to determine the evenness of space use within the home range [12].

To determine habitat associations for each fish we used existing habitat mapping data for Jervis Bay to calculate the proportion of rocky reef and seagrass (predominantly *Posidonia australis*) within the home range and core use areas. To determine activity rates for each fish during the day and night we calculated a Minimum Activity Index (MAI) [36]. This was calculated as the distance moved between waypoints divided by the time elapsed during this movement. Distances between waypoints were calculated using Hawths Tools in ArcGIS v.9.2. MAI represents a conservative value of activity rates as movement between consecutive waypoints was unknown and straight line movement between waypoints was assumed. To determine whether there was a statistical difference in MAI between diel period’s data was analysed based on the mean for each fish using a paired t test.

For the passive tracking data (broad scale movements) we calculated three metrics to give an estimate of site fidelity and movement within the array. Firstly, we calculated a Residency Index (RI) [9], [37] to give an estimate of site fidelity. This was calculated as the number of detection days for each fish on any given receiver divided by the total number of possible detection days, multiplied by one hundred to express as a percentage. For each receiver, as recommended by Vemco, we only used sequences with two or more consecutive detections per day so as to avoid using potentially spurious single temporally isolated detections that may have occurred due to signal collisions or background noise. We made an exception to this rule for one fish (fish 13) after examining the receiver data. Fish 13 appeared to bypass multiple receivers, each receiving a single detection, while making one relatively large trip between reefs and along the coastline to a distant receiver. The time and sequence of detections on receivers indicated this was fish 13’s most likely route. Secondly, the number of receivers (or reefs) visited per day and per week by each fish was calculated to quantitatively describe movements between reefs. Thirdly, we calculated Minimum Linear Dispersal (MLD) [9], defined as the distance between the point of release for each fish and the furthest receiver on which that fish was detected. A distance matrix was generated using ArcGIS v.10 to calculate the distance between all receivers. Linear regression was used to test for a relationship between fish length and both RI and MLD. Activity patterns were assessed by comparing the frequency of detections on each receiver for each hour of the day (24 h). A Rayleigh’s Z test was used to determine whether there was a non-random pattern of detections across the day or diel period (24 h).

### Ethics Statement

This research was approved by the Animal Ethics Committee of the Department of Environment, Climate Change and Water (NSW) (AEC Number: 100802/04). Permission to carry out this research within Jervis Bay was given by the Marine Parks Authority NSW and the Australian Government (BDR10/00006).

## Results

### Fine-scale movements

#### Site fidelity, home range size and core use areas

**Figure 2:**
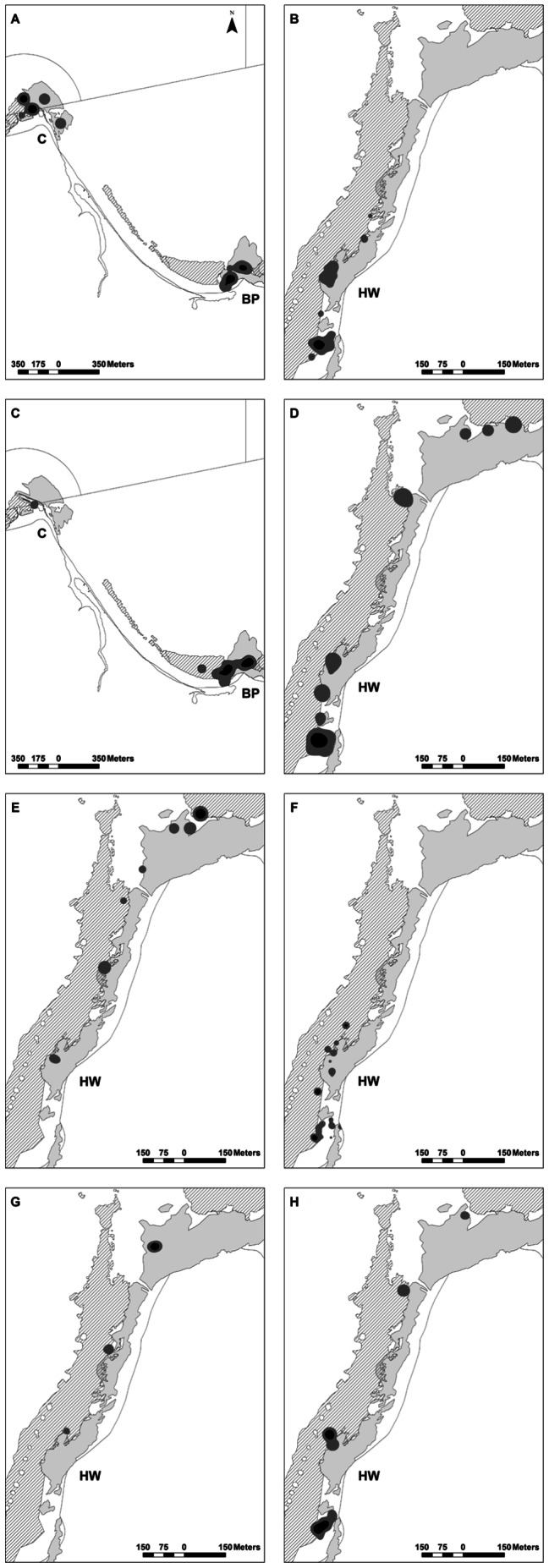
Space utilisation contours for luderick, *Girella tricuspidata*, in Jervis Bay, New South Wales. Core areas (50% usage) are in black and home ranges (90% usage) in dark grey. Panel A – H represents fish 1 – 8 respectively. The site where each fish was caught and released is shown in each panel (BP  =  Bristol Point and HW  =  Hole in the Wall). C (Creswell) is an artificial harbour. Lines in panel A and C represent marine park zone boundaries (see Figure 1). Rocky reef (light grey) and seagrass (hatch) areas are also shown.

**Figure 3:**
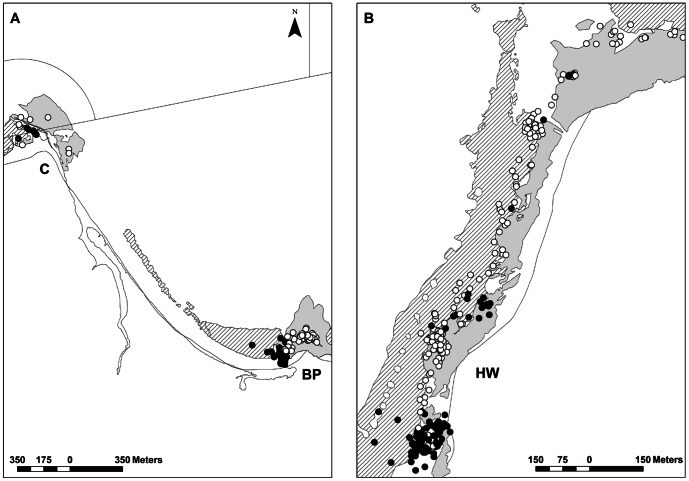
Day (white) and night (black) locations of luderick, *Girella tricuspidata*, in Jervis Bay, New South Wales. Panel A represents pooled locations for fish 1 and 3 and panel B represents pooled locations for fish 2 and fish 4 – 8. Lines in panel A represent marine park zone boundaries (see Figure 1). Rocky reef (light grey) and seagrass (hatch) areas are also shown.

Luderick exhibited strong site fidelity throughout the active tracking period (31 d) (Figs. 2–3, Table 2). All core use areas for each fish were located within 1 km of where they were released, with the exception of fish 1 that used multiple core areas, some ∼2.5 km from its point of release (Fig. 2). Fish were generally detected mainly on the reef where they were caught and released. For the six fish released at Hole in the Wall, all detections were made on or near the continuous stretch of rocky reef that they were released on, although this occasionally involved movements of up to 1 km along this area. We did not observe fish released at Hole in the Wall moving across sand to adjacent reefs >500 m away during the active tracking period. The two fish released at Bristol Point were mainly detected on or near the reef that they were released on, although both made infrequent movements across sand to an adjacent reef (Creswell) ∼2.5 km away, before returning to their release reef (Fig. 2).

**Table 2:** Home range, core area, habitat use and diel activity rates for luderick, *Girella tricuspidata*, actively tracked in Jervis Bay, New South Wales.

									MAI ± SE (m h^−1^)	
Fish ID	Home range (m^2^)	Reef (%)	Seagrass (%)	Core area (m^2^)	Reef (%)	Seagrass (%)	50:90% ratio	# cores	Day	Night
1	76 032	65	7	15 316	58	2	20	4	89±33	54±44
2	11 796	37	31	1 094	0	77	9	1	92±23	43±17
3	55 918	60	17	11 777	94	1	21	2	85±31	30±3
4	26 605	33	37	2 677	0	64	10	1	144±20	47±11
5	8 845	47	39	1 039	0	100	12	1	685±623	24
6	6 212	20	57	670	0	100	11	3	95±27	15±0.16
7	3 298	59	30	664	100	0	20	1	41±18	23±21
8	11 420	14	53	2 629	0	69	23	2	92±23	21

MAI  =  Minimum Activity Index.

All fish, except for two (fish 5 and 8), were detected for >75% of days tracked over the entire active tracking period. All fish except for one (fish 5) were detected on one or both of the last two days of tracking, nearly one month after the active tracking began, and towards the end of the acoustic transmitters battery life (∼37 d) (Table 1). Fish 5 also had the highest daily Minimum Activity Index (MAI) (685±623 m h¯^1^, mean±SE), which was more than four times greater than all other fish (Table 2), indicating that it was moving more frequently and over larger distances compared to the other fish. Importantly, this movement was not unidirectional, but involved frequent trips backwards and forwards along the reef.

Home range size ranged from 3 298 to 76 032 m^2^, with an average home range size of 25 016±9 455 m^2^ (SE) (Fig. 2, Table 2). Core use area within the home range increased significantly (r^2^ = 0.97, df = 7, p<0.0001) with increasing home range size and ranged from 664 to 15 316 m^2^, (4 483±2 025 m^2^, mean ±SE) (Fig. 2, Table 2). The largest home range was up to an order of magnitude bigger than the smallest home range and the largest total core area up to two orders of magnitude bigger than the smallest, however, neither home range size nor total core area were significantly correlated with fish length (r^2^ = 0.02 and 0.05 respectively). Movements were generally concentrated within 1 to 2 core areas, however, two fish (fish 1 and 6) used more than two core areas (Fig. 2, Table 2). The 50:90% ratios were low and ranged from 9 to 23%, (16±2%, mean ±SE), indicating that total core area only made up a small proportion of home range size and that space use within the home range was relatively uneven (Table 2).

#### Habitat associations

Home ranges contained between 14 to 65% shallow subtidal reef (42±7%, mean ± SE) and between 7 to 57% seagrass (34±6%, mean ± SE), predominantly *Posidonia australis* (Table 2). Total core area contained between 0 to 100% (32±16%, mean ± SE) shallow subtidal reef and between 0 to 100% (52±16%, mean ± SE) seagrass, indicating core areas differed largely between fish in the proportion of each habitat type they contained (Table 2). For example, fish 5 and 6 used one and three core areas respectively that all contained 100% seagrass and no rocky reef and fish 2, 4 and 8 also used core areas that predominantly contained seagrass and no reef. In contrast, fish 7 used one core area that contained 100% rocky reef and no seagrass and fish 1 and 3 used core areas that predominantly contained rocky reef and relatively little seagrass (Fig. 2, Table 2).

#### Diel movements

In general, luderick were more active during the day compared to night, moving on average 165±75 m h¯^1^ (SE) during the day compared to 32±5 m h¯^1^ (SE) at night (Table 2). There was, however, large variation in daytime movements of fish making it difficult to detect a significant difference in MAI between diel periods (t = 1.76, df = 7, p = 0.06). Luderick exhibited predictable patterns in movement between spatially distinct daytime and night-time core use areas and multiple fish also shared a similar core location during the day as well as at night (Figs. 2–3). For example, fish 2, 4, 6 and 8 all shared a similar core location during the night, (located at the southern end of the reef at Hole in the Wall), where they remained relatively inactive, before moving to other core areas or activity centres used during the day, and then returned to their shared core location at night (Figs. 2–3). Likewise, fish 1 and 3 both shared a similar core location during the night, (located at the southern end of the reef at Bristol Point), where they remained relatively inactive, before moving ∼100 m north-east to a separate core location which they also shared during the day (Figs. 2–3). Fish 1 also used a second core area on some nights, located at Creswell ∼2.5 km from the reef where it was released (Figs. 2–3). Core areas occupied by fish at night were generally characterised as sheltered coves situated on the edge of the reef least exposed to the prevailing wind and swell conditions, and in the case of fish 1 the second core area used on some nights was located behind a large (∼200 m) breakwall within a small artificial harbour at Creswell (Figs. 2–3).

### Broad scale movements

#### Site fidelity

**Figure 4:**
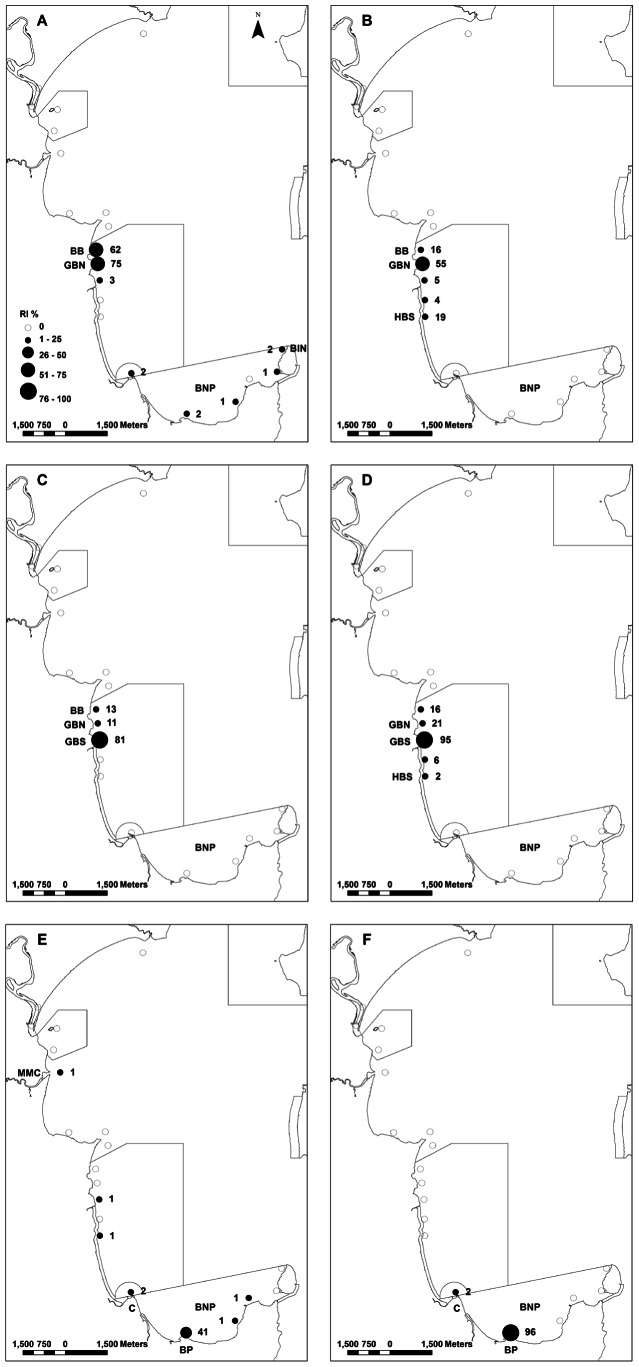
Site fidelity of luderick, *Girella tricuspidata*, passively tracked in Jervis Bay, New South Wales. Panel A – F represents fish 9 – 14 respectively. The site where each fish was caught and released is shown in each panel (GBN  =  Greenfields Beach North, GBS  =  Greenfields Beach South and BP  =  Bristol Point) (refer to Table 1). BB (Blenheim Beach), BIN (Bowen Island North), HBS (Hyams Beach South), MMC (Moona Moona Creek) and C (Creswell) all represent other sites visited by fish (refer to Table 3). Boxed areas in panels represent marine park zone boundaries (see Figure 1). Graduated symbols and values represent Residency Indexes (RI) i.e. number of days detected on each receiver divided by total number of possible detection days, multiplied by 100 to express as a percentage. Open circles represent receivers with no detections.

**Figure 5:**
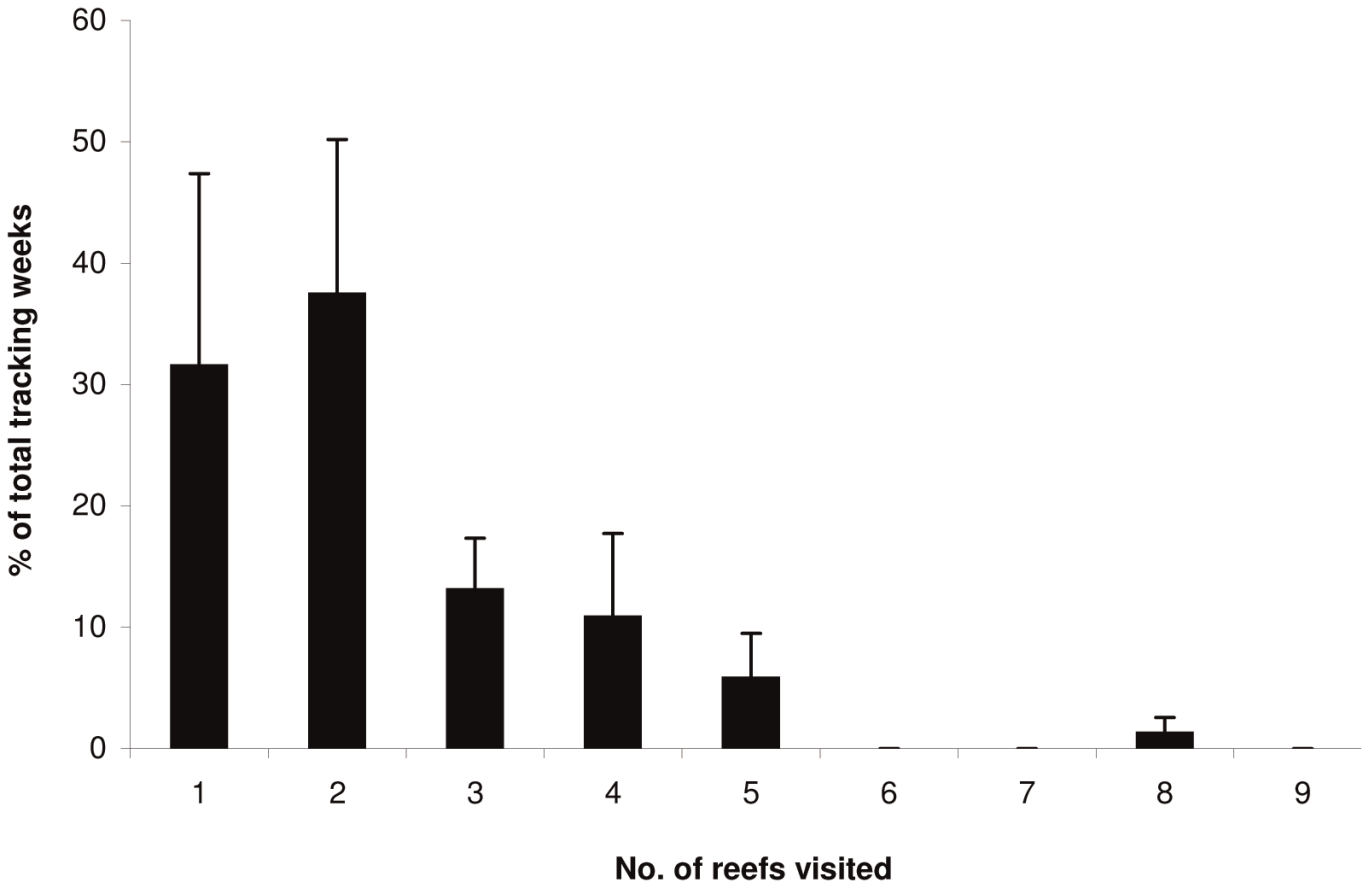
Number of reefs visited per week by luderick, *Girella tricuspidata*, expressed as a percentage of total weeks passively tracked. Percentages are presented as the average across the six fish passively tracked over a period of ∼14 weeks.

Luderick passively tracked exhibited strong site fidelity (Fig. 4). For each of the six fish, the Residency Index (RI) was highest at the site in which they were caught and released with, on average, fish being detected on ∼74% of days over the three month tracking period on the reef in which they were released (Fig. 4). Two of the six fish (fish 12 and 14) were even detected on the reef that they were released on for ≥ 95% of days (although both made sporadic trips to neighbouring reefs). Although most fish appeared to reside on their release reef many did make frequent trips between their release reef and adjacent reefs (Figs. 4–5, Table 3). The median number of reefs visited per day by fish ranged between one and two (Table 3). The median number of reefs visited by fish per week did not change from the number of reefs visited per day for all but two fish (fish 10 and 11) (Table 3). Fish 10 visited a median number of four reefs per week (Table 3). On average, most fish visited only two reefs per week and visits to more than four reefs per week were rare (Fig. 5). These reasonably small movements between reefs across 200–300 m of sand were the most common trips that fish made and they generally returned to their release reef within a relatively short period of time. Even after relatively large trips this was observed to occur. For example, fish 9 and fish 13 made trips of at least 7.8 and 10.8 km respectively, and then returned to their release reef within 2.5 and 3 d respectively.

**Table 3:** Description of movements between rocky reefs by luderick, *Girella tricuspidata*, passively tracked in Jervis Bay, New South Wales.

	Median # of reefs visited					
Fish ID	Per day	Per week	MLD (km)	Catch-release reef	Commonly visited reef	Furthest visited reef
9	2	2	7.8	GBN (sz)	BB	BIN
10	1	4	2.4	GBN (sz)	BB	HBS
11	1	2	1.1	GBS (sz)	GBN	BB
12	2	2	2.4	GBS (sz)	GBN	HBS
13	1	1	10.8	BP (BNP)	C	MMC
14	1	1	2.4	BP (BNP)	C	C

MLD  =  Minimum Linear Dispersal. GBN  =  Greenfields Beach North, GBS  =  Greenfields Beach South, BP  =  Bristol Point, BB  =  Blenheim Beach, BIN  =  Bowen Island North, HBS  =  Hyams Beach South, MMC  =  Moona Moona Creek, C  =  Creswell. BNP  =  Booderee National Park, sz  =  sanctuary zone (no fishing allowed).

Of the four fish released within the sanctuary zone, only one fish (fish 9) was detected moving outside the sanctuary zone during one relatively large trip. Despite making this trip over a relatively large distance it returned to the reef that it was released on within a few days (Fig. 4, Table 3). Of the two fish caught and released outside the sanctuary zone (fish 13 and 14), both travelled over similar spatial scales to those fish tagged within the sanctuary zone (Fig. 4, Table 3). One travelled only to Creswell, while the other ranged widely along the shore of the bay (Fig. 4, Table 3). Both returned to Bristol Point, with one fish remaining there during the entire tracking period, while the other was lost from the array after ∼41 d (Table 1). The movements of the two fish released at Bristol Point were consistent with those of fish 1 and 3 (also caught and released at Bristol Point) during the active tracking (Fig. 2 and 4). These fish all moved from their release reef (Bristol Point) to Creswell, ∼2.5 km away, before returning to their release reef.

Minimum Linear Dispersal (MLD) ranged from 1.1 to 10.8 km (4.5±1.6 km, mean ± SE) (Table 3). Fish 13 had the highest MLD and was also one of two fish not detected within the array for >75% of days tracked, its last detection being ∼1 month before the end of the passive tracking period (Table 1 and 3). Neither RI nor MLD were significantly correlated with fish length (r^2^ = 0.08 and 0.09 respectively).

#### Diel movements

**Figure 6:**
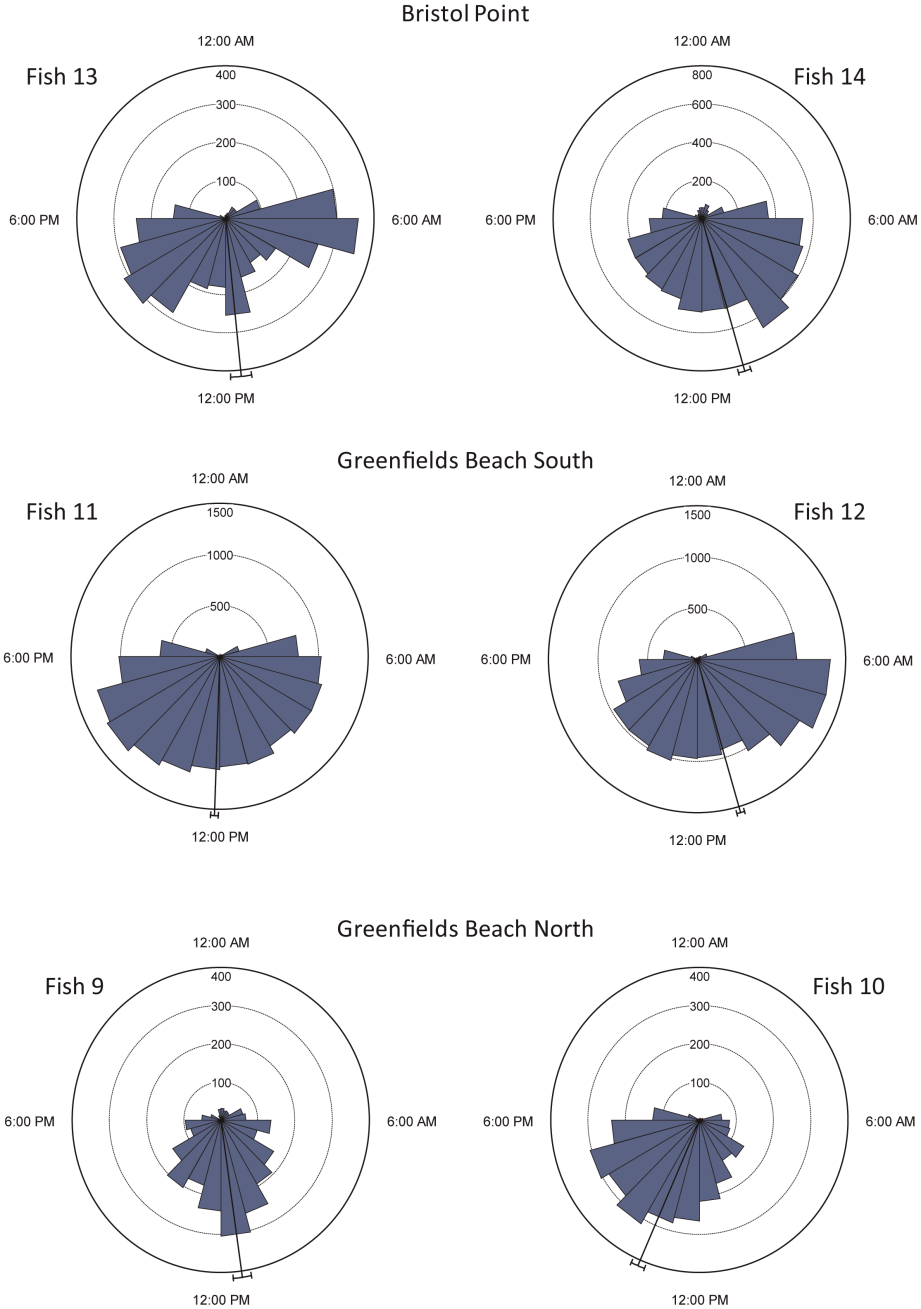
Circular histograms showing diurnal frequency of detections on acoustic receivers for luderick, *Girella tricuspidata*, passively tracked in Jervis Bay, New South Wales. The mean period of detections and 95% confidence intervals are shown. Fish are paired together based on the location that they were caught and released, and where they spent most time during the study.

Luderick clearly showed a non-random pattern of detections, with detections being more frequent during the day (0601 to 1800 h) compared to night (1801 to 0600 h) (Fig. 6, Table 4). The pattern of detections was consistent with what we would have predicted based on the fine-scale movement data. During the active tracking we observed that luderick sheltered on the protected (i.e. least exposed) edges of reefs at night. As the receivers were deployed at the midpoint of reefs, rather than on the edge of reefs, the receivers would have been unlikely to detect fish sheltering in these areas, due to the reef edge obstructing the transmitter signal. Generally, we found that initial detections corresponded with sunrise and final detections with sunset (Fig. 6). The difference in the frequency of detections on receivers between the day and night was not due to a reduction in the effectiveness of receivers to detect unobstructed transmitter signals at night, as detections of the reference transmitters (deployed for 7 days and nights at 2 representative reefs) were only reduced by 1.2% and 2.6% at night at Greenfields Beach North and Bristol Point respectively. The average number of detections between day and night at Greenfields Beach North was 233 h¯^1^±2.28 (SE) and 227 h¯^1^±2.9 (SE) respectively, and the average number of detections between day and night at Bristol Point was 246 h¯^1^±1.08 (SE) and 243 h¯^1^±2.22 (SE) respectively. The maximum number of detections of unobstructed signals from reference transmitters would be expected to be ∼270 h¯^1^ (Vemco, personal communication). As the difference in the frequency of detections between day and night for all fish was far greater than the difference in frequency of detections of the reference transmitters between day and night, it indicates that differences in detectability between diel periods was not an artefact of receiver effectiveness, but rather due to the movements of fish.

**Table 4:** Summary statistics of diurnal frequency of detections on acoustic receivers for luderick, *Girella tricuspidata*, passively tracked in Jervis Bay, New South Wales.

Fish ID	Number of detections	Mean of detections	Circular standard deviation	Length of mean vector (r)	Rayleigh Z-test (*p*)
9	2 460	11:28	4:07	0.558	<0.001
10	2 732	13:34	3:31	0.653	<0.001
11	15 257	12:07	4:12	0.544	<0.001
12	13 772	10:54	4:08	0.557	<0.001
13	3 471	11:36	4:58	0.429	<0.001
14	6 912	10:55	4:30	0.498	<0.001

## Discussion

The ecologically important herbivore luderick, *Girella tricuspidata*, exhibited strong site fidelity on shallow subtidal reefs within this temperate marine park and used discrete core areas consistently during the day and night. Movement patterns and behaviour of luderick observed here indicate a high level of habitat familiarity, and suggests that fish were residing on release reefs. Luderick are clearly capable of moving large distances along the coastline [27], [32], [33], and some fish were observed making relatively large trips between reefs in this study, however the majority of their time was spent on what we would argue are “home” reefs (i.e. release reefs). Fish made trips from these home reefs, but generally these were to adjacent reefs and not much further. When fish did move beyond the next adjacent reef they usually returned to their home reef on the same day, or within a few days.

Fine scale habitat use on home reefs was sophisticated and consistent. Active tracking revealed luderick occupied regular core areas during the night, behind the edge of reefs in sheltered coves on adjacent soft sediment or within seagrass beds, where they remained relatively inactive. During the day luderick moved to discrete core areas on home reefs, most likely to forage, and also visited other nearby reefs. Passive tracking supported the observations made during the active tracking, with behaviour of luderick appearing to be relatively consistent over time. These observations were surprising as few studies have described this level of sophisticated interaction between fish and the specific reefs that they inhabit [38].

Despite observing consistent patterns in habitat use and site fidelity by luderick we acknowledge that the data presented here are preliminary. We tracked fourteen fish over a four month period (active and passive tracking combined). Nevertheless, the data presented here represent two independent sampling periods and two independent groups of fish tagged at multiple independent sites, showing consistent patterns in movement and behaviour. Furthermore, the time of year that passive tracking took place (i.e. December to March) covers much of the spawning period of luderick (October to January in southern NSW) [27] and all fish passively tracked, based on their length, would have attained reproductive maturity [27]. We expected that due to this some fish would have left the array, as fish migrated to spawning areas, and that this could potentially have provided us with an estimate of the relative numbers of fish that make spawning migrations as well as migratory routes along the coastline (determined from detections on receivers deployed along the NSW coastline). Almost all tagged fish, however, remained within the array on home reefs over the entire tracking period. Clearly, tracking more fish and over longer periods of time will be essential in order to provide a greater understanding of the behaviour of luderick and this initial investigation forms part of a longer term assessment of movement patterns for this species. Nevertheless, incremental reporting as new developments in our understanding occur is valuable and findings should be disseminated.

Our observations of residency within an embayment correspond with previous mark-recapture studies showing that the majority of tagged luderick were recaptured in the estuary in which they were released [27], [32], [33]. Our main contribution here, however, is to demonstrate fine scale habitat use of luderick and frequency of movements between reefs. Gray et al. [27] suggested that because the majority of recaptures of luderick within the same estuary were within a short time period (although it is unclear what these time periods were) that this may not indicate permanent residency. Nevertheless, in some instances luderick have been recaptured up to two years later in the same estuary in which they were released [32], indicating that some fish probably reside within estuaries for long periods of time. Acoustic telemetry allows researchers to determine fine scale differences in residency behaviour not possible with mark-recapture experiments and if receivers are deployed in and across the entrance to estuaries (as is the case for many estuaries in NSW) and along the coastline then movements within and between estuaries can be accurately assessed. The combination of using active and passive tracking also appears to be rare (18), however the information that can be gained and used to independently assess or corroborate apparent patterns from each technique highlights the significant value of this approach.

The apparent site fidelity observed in our study (over a four month period) and that observed over longer term tagging studies [27], [32], [33] is difficult to reconcile with the obvious spawning migrations that luderick make [27], [31]. A possible explanation is that luderick exhibit partial migration. Morrison [39] suggested this for luderick after observing what appeared to be concurrent resident and migratory groups on reefs within the population he studied. In the current study the two fish that went missing had the highest MAI and MLD of all other fish tracked and partial migration may explain why these two fish were unable to be located at the end of the active and passive tracking periods, as it is possible they migrated out of the array or sampling area. It is also possible they were simply eaten by predators, caught by anglers, or died of another cause. If luderick do exhibit partial migration then those that are resident would be expected to benefit most from protection provided by sanctuary zones, and those that are migratory would be expected to benefit least, as they have increased exposure to fishing outside sanctuary zones. Biro and Post [40] showed that the behaviour of individual fish (e.g. activity, boldness) can be selected against by exploitation. In light of the present study carried out here and previous mark recapture studies [27], [32], [33], the weight of evidence suggests that the majority of luderick exhibit some degree of site fidelity and could benefit from marine protection. Furthermore, if our initial observations on the behaviour and movement patterns of luderick are found to be consistent over time (which is currently being assessed) it would suggest that sanctuary zones within marine parks covering one reef or a system of reefs may provide significant protection for fish inhabiting those reefs. Considering that luderick are relatively long lived (>25 yrs) [27] long-term site fidelity may also lead to significant increases in size and abundance within sanctuary zones if the rate of natural mortality is considerably lower than fishing mortality.

There was a disproportionate use of space by luderick within each home range, as indicated by the 50:90% ratio. While luderick were observed ranging over a relatively large area, generally half of their time was spent in core use areas that made up less than a quarter of their total home range size. Some areas within the home range probably provided better shelter or foraging areas than others, so it is logical that luderick would spend more time in certain areas than others. Core use areas were consistent with locations where luderick sheltered during the night. In general, fish released at the same site “nested” within close proximity to one another during the night in sheltered, core locations, before moving to other core areas and activity centres used during the day. This observed pattern in diurnal movements correlates well with diel feeding patterns observed in many species of marine herbivorous fishes, whereas feeding activity is greatest during the day, peaking in the afternoon, before decreasing prior to sunset [41].

Habitat use and diel movement patterns of luderick observed during the active tracking appeared to be consistent with those observed during the passive tracking. During the passive tracking luderick were detected on receivers far more frequently during the day than at night and the patterns of detections appeared to be associated with sunrise and sunset. This pattern was predicted *a priori* based on our observations of luderick behaviour made during the active tracking and also based on the position of receivers on each reef. During the active tracking luderick sheltered behind the edge of reefs at night and remained relatively inactive within small core areas. Our positioning of receivers at the midpoint of reefs meant that we were confident of tracking movements of fish along and between reefs, however we would be unable to detect fish when they sheltered at night as receivers would have been obstructed from detecting signals by the reef edge. To cover these night-time sheltering areas behind the reef edge, as well as broad areas of the reef and adjacent shoreline, was beyond the scope of the current study due to the large number of receivers which would have been required. Nevertheless, the pattern of detections fits well with our prediction of far less detections at night due to nocturnal sheltering. Fish may have been active in areas behind the edge of reefs at night, however this is unlikely as sheltered areas behind the edge of reefs were relatively small and fish would only have had to move a relatively small distance before they were within range of receivers again. If fish were highly active at night on the reefs during the passive tracking we are confident we would have detected them. In general, reefs were low relief with only relatively small areas of complexity and few obstructions to receiver signals, while receiver coverage was relatively extensive (i.e. covering >400 m of the shoreline at most reefs and extending out from the shore to past the receiver itself). Additionally, we were able to rule out a reduction in the effectiveness of receivers to detect unobstructed transmitter signals at night, as detections of reference transmitters differed little between day and night. Hence, overall we are confident that the passive tracking data indicate that differences in patterns of detection and habitat use between day and night were temporally consistent with diel movement patterns observed during the active tracking.

Core areas used during the day were either dominated by rocky reef or seagrass (predominantly *Posidonia australis*). Kingsford [24] observed large numbers of luderick foraging on shallow rocky intertidal reefs in Jervis Bay, grazing on turfing algae where the chlorophyte *Ulva* spp. covered a large proportion of the substratum. We have previously observed, on multiple occasions, large numbers of luderick grazing patches of turfing algae from reefs at the same location as core use areas identified during the active tracking. A disproportionate amount of foraging may have been occurring within these core areas and some individuals may have had preferential foraging grounds. Core use areas located on reefs and occupied during the day also corresponded with small areas of structural complexity i.e. boulders, overhangs, small caves and crevices, and may have been used as shelter. Welsh & Bellwood [42] found that the herbivorous parrotfish, *Chlorurus microrhinos*, used core areas that corresponded with a greater number of feeding scars and greater topographic complexity and suggested that these areas were selected for grazing and because of decreased predation risk. Seagrass also appeared to be important habitat used by luderick, in particular for shelter and potentially resting as fish remained relatively inactive in these areas during the night. During the day much of the activity was concentrated along the reef edge which fringed large seagrass beds at some sites (e.g. Hole in the Wall). This may have overestimated the importance of seagrass use during the day, as fish were rarely detected far out into the seagrass, but rather on the edge or fringe of reefs during the day.

The movement patterns of luderick observed here fits with other temperate reef fishes that have been shown to exhibit strong site fidelity on reefs, including sparids [11], [12], [13], [43], labrids [37], [44], cheilodactylids [45], monocanthids [44] and sebastids [38], [46]. The generality of this pattern amongst temperate reef fishes [47], covering a range of families and diverse functional roles, emphasise the potential value of sanctuary zones in protecting diverse assemblages of fish. Rarely have studies assessed the movements of herbivores, and this should be addressed given their large biomass on reefs [19] and potentially important role in ecosystem function [19], [48], [49] and how this relates to the functioning of marine parks. Considering the generally small scale movements of luderick in this study it should be possible to assess the ecological function provided by this species and how this may be influenced by protection within marine parks.

In conclusion, our data indicate strong site fidelity and consistent use of home reefs and night time sheltering areas by luderick and, taken in conjunction with previous tagging studies, suggest that luderick may respond positively to protection provided by sanctuary zones. Of course, other factors will be important in determining the likelihood of this outcome (e.g. the level of fishing effort in surrounding areas, compliance, recruitment success and other large-scale threats including pollution and climate change), although based on the movement patterns of luderick determined in this study the current zoning plan for Jervis Bay Marine Park appears to be appropriate, in terms of the spatial extent of sanctuary zones within the marine park. This study also highlights the benefit of combining both active and passive acoustic telemetry techniques in order to unravel some of the complexities and uncertainty in movement patterns within a species. If we are to fully understand the complexities of fish movements and appropriately assess the effects of marine protection there is a need to critically evaluate conventional views of fish movements we may have through quantitative assessment of movement patterns at the correct spatial and temporal scales.
